# Hypersensitivity and Induced Radioresistance in Chinese Hamster Cells Exposed to Radiations with Different LET Values

**DOI:** 10.3390/ijms23126765

**Published:** 2022-06-17

**Authors:** Ekaterina Koryakina, Vladimir I. Potetnya, Marina Troshina, Raisa Baykuzina, Sergey Koryakin, Anatoliy Lychagin, Aleksei Solovev, Vyacheslav Saburov, Vladimir Pikalov, Petr Shegay, Sergey Ivanov, Andrey Kaprin

**Affiliations:** 1 A. Tsyb Medical Radiological Research Center, Branch of the National Medical Research Radiological Center of the Ministry of Health of the Russian Federation, 249036 Obninsk, Russia; potetnya@yandex.ru (V.I.P.); troshina-m-v@mail.ru (M.T.); baykuzina_rm@mail.ru (R.B.); korsernic@mail.ru (S.K.); lychagin1@yandex.ru (A.L.); salonf@mrrc.obninsk.ru (A.S.); vosaburov@gmail.com (V.S.); oncourolog@gmail.com (S.I.); 2 Institute for High Energy Physics Named by A. A. Logunov of National Research Center «Kurchatov Institute», 142280 Protvino, Russia; vladimir.pikalov@ihep.ru; 3 National Medical Research Radiological Center of the Ministry of Health of the Russian Federation, 246036 Obninsk, Russia; dr.shegay@mail.ru (P.S.); kaprin@mail.ru (A.K.); 4 Federal State Autonomous Educational Institution of Higher Professional Education “Peoples’ Friendship University of Russia”, Medical Institute, Department of Oncology and Radiology Named after N.P. Kharchenko, 117198 Moscow, Russia

**Keywords:** hyper-radiosensitivity, induced radioresistance, chromosome aberrations, G1 phase, Chinese hamster ovary cells, protons, neutrons, carbon ions

## Abstract

We study the impact of radiation LET on manifestation of HRS/IRR response in Chinese hamster cells ovary cells exposed to radiations used in radiotherapy. Earlier we have investigated this response to carbon ions (455 MeV/amu) in the pristine Bragg curve plateau and behind the Bragg peak, ^60^Co γ-rays, and 14.5 MeV neutrons. Now we present results of cytogenetic metaphase analysis in plateau-phase CHO-K1 cells irradiated with scanning beam protons (83 MeV) at doses < 1 Gy and additional data for 14.5 MeV neutrons. Dose curves for frequency of total chromosome aberrations (CA, protons), paired fragments (protons, neutrons), aberrant cells (neutrons) had typical HRS/IRR structure: HRS region (up to 0.1 and 0.15 Gy), IRR region (0.1–0.6 Gy and 0.15–0.35 Gy) for protons and neutrons, respectively, and regular dose dependence. Taken together with previous results, the data show that LET increase shifts the HRS upper border (from 0.08–0.1 Gy for γ-rays, protons and plateau carbons to 0.12–0.15 Gy for “tail” carbons and neutrons). The IRR regions shortens (0.52–0.4 γ-rays and protons, 0.25 plateau carbons, 0.2 Gy “tail” carbons and neutrons). CA level of IRR increases by 1.5–2.5 times for carbons as compared to γ-rays and protons. Outside HRS/IRR the yield of CA also enhanced with LET increase. The results obtained for different LET radiations suggest that CHO-K1 cells with G1-like CA manifested the general feature of the HRS/IRR phenomena.

## 1. Introduction

In 1993, Marples and Joiner discovered a new, low-dose phenomenon using the survival response of V79 hamster fibroblasts to single X-ray doses [1], termed later HRS/IRR. “Low dose hyper-radiosensitivity (HRS) is characterised by an increased sensitivity to radiation doses less than 0.3 Gy, which is followed by a more radioresistant response per unit dose between 0.3 and 0.6 Gy termed increased radioresistance (IRR)” [2] (p. 98). This phenomenon has been later demonstrated in numerous studies on mammalian cell survival, chromosomal aberration, and micronucleus induction in mammalian and plant cells, and also using DNA double-strand break (DSB) test. It was shown that not all types of cells were capable of exhibiting the effects. Only 75% of the 50 mammalian normal and malignant cell lines tested by 2010 using a clonogenic assay in vitro exhibited the HRS/IRR response [2]. Some high-LET radiations did not trigger this effect.

Many hypotheses exist on mechanisms of HRS and IRR, starting from the assumption that HRS/IRR, or rather the specific pattern of the initial part of dose curves, is exhibited in the cell population, which was a mixture of cells differing in radiation sensitivity (e.g., [3]). The main problem was the mechanism of HRS, while as far as IRR was concerned, it was agreed that this cellular response resulted from radiation damage repair induction. It was hypothesised that HRS was due to the presence of G2-phase cells subpopulation in the cell culture, or due to apoptotic death of damaged cells to prevent mutation perseverance in survived cells. The latter was later invalidated by observing the HRS response in mammalian cells using chromosomal and gene mutation assays [4]. Other possible mechanisms of low-dose HRS that have been investigated include impairment of DNA damage repair, the impact of cell cycle checkpoints, DNA DSB repair pathways and their regulation, as well as NO-mediated cell death. Following a series of studies [5] using clonogenic, micronucleus induction, γH2AX, and pATM foci assays, researchers have concluded that HRS was observed in primary normal fibroblasts in both asynchronous and G2-phase cells of HRS-positive patients, while it was absent in cells from HRS-negative patients. Enrichment of the population with G2-phase cells has been shown to have no effect on eliciting HRS, though the “HRS response in these cells is associated with the functioning of early G2-phase checkpoint in a threshold-dose dependent manner, similarly as it takes place in most of human tumour and other cells” [6] (p. 45). Wang et al. [7] suggested that early G2-phase checkpoints play important roles in the induction of the DNA damage repair and IRR after threshold doses of 0.2–0.3 Gy.

According to a current radiobiological paradigm, the DNA double-strand breaks are the major cellular damage that results in cell death, mutations, and chromosomal aberrations (CA). In this regard, radiations with different LET values are a helpful tool to investigate the influence of DSB complexity on various radiobiological effects manifestation using the above endpoints because the DSB complexity increases with LET. It should be also noted that the typical dose range of HRS is up to 0.1–0.2 Gy, i.e., it is within the small dose range considered in radiation protection [8], where stochastic rather than deterministic effects prevail. At these doses, the mean specific energy z in a 8 μm cell nucleus becomes constant at doses <2.5 and <73 mGy for ^60^Co γ-rays and 14 MeV neutrons, respectively. After this kind of threshold, the mean elemental dose begins to rise proportionally to the absorbed dose [9]. However, the fractions of cells hit increase with a dose below those thresholds and reach 100% above them. The above micro-dosimetric considerations raise the question of an HRS threshold for radiations differing in LET levels.

The HRS survival response has been observed after exposure to some high-LET radiation: pi-meson and proton [10,11,12,13], as well as 14 MeV neutrons given at a low dose rate [14]. On the basis of experiments with pi-mesons in the Bragg peak and d(4)-Be fast neutrons, the authors of [11,12,13] suggested that the IRR response was only evident after low and intermediate LET radiation exposures. Experiments with 59 and 79 keV/μm α-particles allegedly confirmed this suggestion, but those with 102 keV/μm α-particles did not [15]. Results of experiments with carbon ions in which LET levels were 45.2 keV/μm [16], 70 keV/μm [17,18], and 252 keV/μm [19] also disagreed with this suggestion. Our cytogenetic studies with CHO-K1 cells exposed to low-dose rate 14.5 MeV neutrons have also shown HRS/IRR response [20], as well as in B14–150 cells irradiated with carbon ions at the plateau and “tail” of the Bragg curve [21]. Both cell cultures were in the stationary (plateau) growth phase.

Most of the results on HRS/IRR in vitro were obtained using asynchronous cell populations and clonogenic assay, with an emphasis on G2-phase cells where the effect was most readily expressed. However, G1-phase cells also showed the effect [22]. It is known that cells irradiated in G0 or G1 phases die predominantly due to the visible Giemsa-stained chromosome-type CA dicentrics, centric and acentric rings, and terminal deletions. Cornforth and Bedford established a one-to-one relationship between the average number of these aberrations per cell and −ln *S*, where *S* is the fraction of surviving cells [23]. The fact allows for comparing results of HRS/IRR response cytogenetic studies with those obtained using survival assays.

In this paper, we present the results of cytogenetic studies with Chinese hamster ovary cells exposed to protons and 14.5 MeV neutrons and compare them with previous data obtained using neutrons, carbon ions, and γ-rays. Taken together, the data show a certain dependence of HRS/IRR response on the LET and in general agree with the assumption that it is induced by low and intermediate LET radiations.

## 2. Results

Cells were irradiated in the late stationary growth phase. Therefore, chromosome-type aberrations (paired fragments, dicentrics, centric and acentric rings) contributed mainly to the total CA yield.

### 2.1. Cytogenetic Effects of Protons at the Bragg Curve Plateau

**Figure 1:**
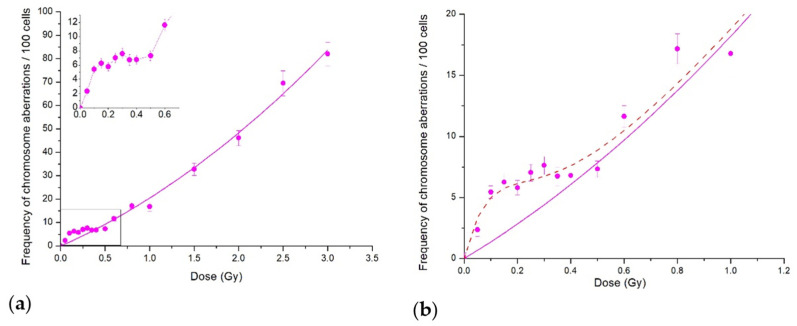
Cytogenetic effects of protons at the Bragg curve plateau for CHO-K1 cells: (**a**) CA in the whole dose range studied; the inset shows the low-dose region; (**b**) CA for doses below 1 Gy. Each point represents the mean ± SEM. Solid lines are LQ model approximations; dash line shows our fit to the data of the IR model.

The dose–response curves for the total frequency of CA induced in CHO-K1 cells via the scanning proton beam irradiation are presented in Figure 1. The protons LET was estimated to be ≈1 keV/μm.

If we consider the dose curve in the whole dose range studied up to 3.5 Gy (Figure 1a), the experimental data fit well with the linear–quadratic model. However, the initial part of the curve shown in the inset demonstrates clearly the irregular behaviour in terms of the standard linear–quadratic model. The induced-repair model (IR model) of Marples and Joiner [1] readily describes the data (Figure 1b). The HRS region where CA frequency increased sharply was at doses < 0.1 Gy, and the IRR plateau lay in the dose range of 0.1 to 0.5 Gy. Further dose increase returned the dose curve back to the linear–quadratic model. Similar dose curve patterns were obtained for the frequency of paired fragments, which were the most common type of aberrations (data not shown) in plateau-phase CHO-K1 cells.

**Figure 2:**
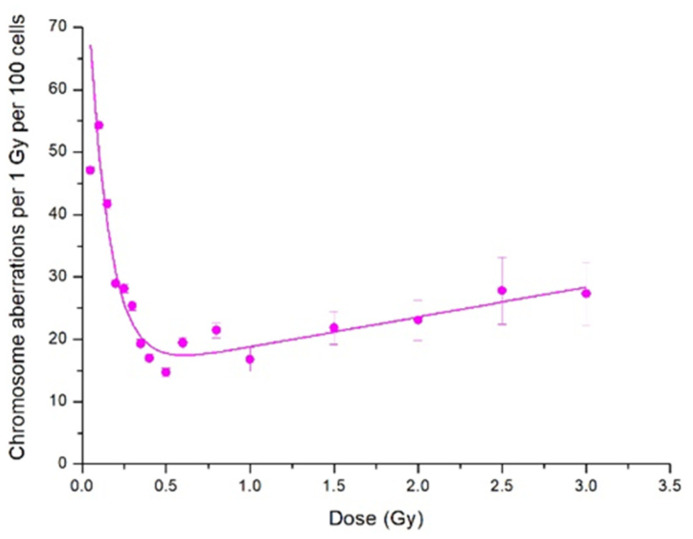
Yield of CA in CHO-K1 cells normalised per proton dose. Points are experimental data, and lines show our fits to the IR model.

Another evidence of the HRS and IRR response is the dependence of radiosensitivity, i.e., CA frequency per unit dose, on absorbed proton dose. Figure 2 shows the same experimental data as those presented in Figure 1 but normalised per proton doses. The general view of Figure 2 reveals two distinct areas—one with fast decreasing radiosensitivity and the other with its slow increase. The slowly increasing one corresponds to the βD term of normalised LQ dependence, while interception of its extrapolation with the *y*-axis corresponds to the linear slope of the dependence.

Two important features follow from Figure 2. Firstly, the radiosensitivity maximum was at doses at which CA frequency showed HRS response. Secondly, the increased radiosensitivity persisted in the dose range of IRR response, although decreasing in value. It reached the level predicted with the LQ model (≈15 CA per 1 Gy per 100 metaphases) at doses of transition to LQ dose dependence. We emphasise once more that increased radiosensitivity extends beyond the range of HRS response, and this observation is relevant in radiation protection.

### 2.2. Cytogenetic Effects of 14.5 MeV Neutrons

**Figure 3:**
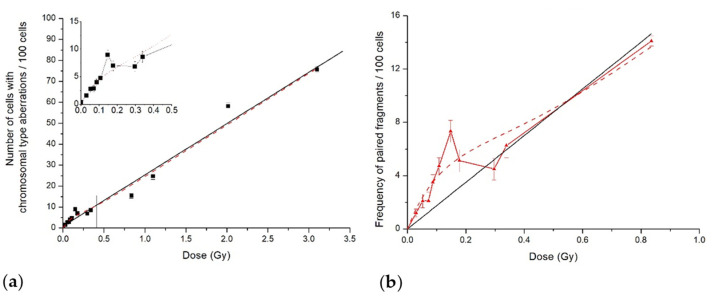
Cytogenetic effects in CHO-K1 cells induced by 14.5 MeV neutrons: (**a**) number of cells with aberrations of chromosome type; (**b**) frequency of paired fragments. Symbols are experimental data; solid lines are LQ model approximations, whereas dash line shows our fit to the data of the IR model.

Data for total chromosome aberrations frequency induced in CHO-K1 cells by 14.5 MeV neutrons were published earlier [20]. Here, we demonstrate the initial part of the dose curve for the number of cells with all chromosome-type aberrations, as well as that for paired fragments frequency (Figure 3). Cells with chromosome-type aberrations are doomed to die [23], and their frequency is an estimation of cell reproductive death. Unlike low-LET protons, neutrons are high-LET radiation with a dose-averaged LET_d_ of 100 keV/μm. This LET difference results in the difference between dose curves: linear–quadratic for protons (Figure 1) and linear for neutrons (Figure 3; Figure 2 in [20]).

**Figure 4:**
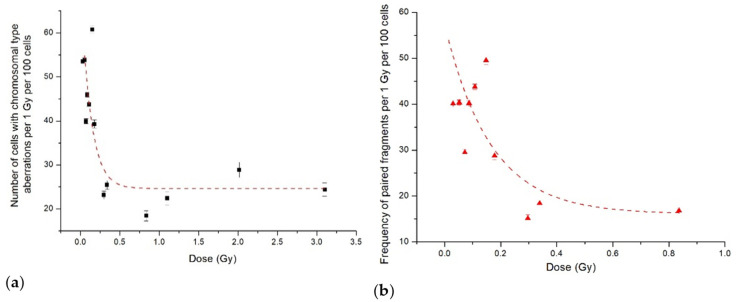
The data from Figure 3 normalised per dose: (**a**) number of CHO-K1 cells with aberrations of chromosomal type; (**b**) frequency of paired fragments. Symbols are the experimental data, and lines show our fits to the IR model.

However, as in the case of proton irradiation, there was HRS/IRR response both for the percent of cells with chromosome-type aberrations and paired fragments’ frequency following 14.5 MeV neutron irradiation. The hyper-radiosensitivity was observed at doses up to 0.15 Gy, and the induced radioresistance was in the dose range of 0.15–0.35 Gy. Experimental results normalised per absorbed neutron dose also clearly demonstrated high radiosensitivity in the HRS dose range, followed by its decrease in the IRR region (Figure 4).

### 2.3. HRS/IRR Response following Exposure to Radiation of Different Quality in the Low Dose Range

**Figure 5:**
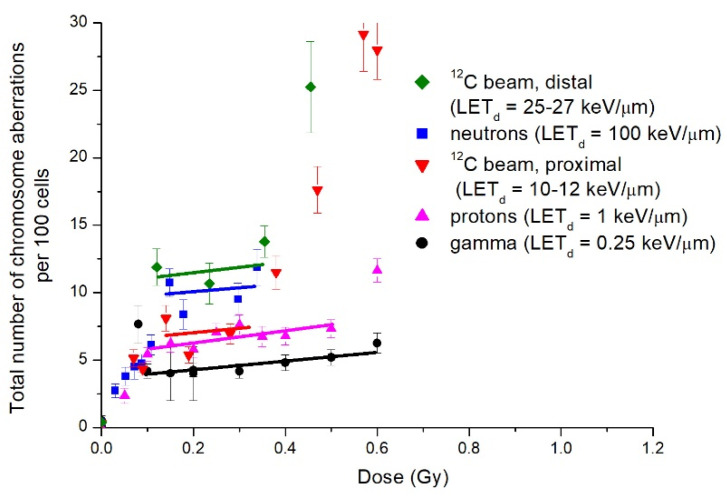
Total frequency of chromosome-type aberrations induced by radiations with different LET values. Legend lists radiations in the order of chromosome aberration level in the IRR response dose range increase. In parentheses: dose-averaged LET_d_.

The above cytogenetic data obtained for radiations with very different LET values, 1 or 100 keV/μm, supplemented the results of our previous studies using the same cell line exposed to ^60^Co γ-rays and carbon ions [20,21,24] of intermediate LET values. Detailed cytogenetic results of all experiments are presented in Table A1, Table A2, Table A3, Table A4 and Table A5 of Appendix A. Figure 5 aggregates the initial parts of dose curves for radiations studied. Solid lines pass through data points in IRR dose ranges. Data for 14.5 MeV neutrons, presented in Figure 5, are from a previous study [20].

Before summarising the data shown in Figure 5, we briefly remind general findings from previous studies. Dose curves for total CA and paired fragments frequencies in plateau-phase CHO-K1 cells, exposed to γ-rays and carbon ions, had an irregular shape at doses below 1 Gy, indicating the HRS and IRR response. The IRR plateaus were observed at 0.08–0.6 Gy for γ-rays and at 0.15–0.35 Gy for carbon ions, the average CA frequency being 1.5–2.5 times higher for ions compared with that of γ-rays.

Three major conclusions ensue from Figure 5. Firstly, the upper doses of HRS response were within 0.08–0.15 Gy, and the dose curve slopes were roughly equal. Secondly, IRR response dose range, in general, decreased with LET increase. It was the largest for ^60^Co γ-rays, 0.08–0.6 Gy, less for protons, 0.1–0.5 Gy, and the least for carbon ions and 14.5 MeV neutrons, from (0.12–0.15) to 0.35 Gy. Thirdly, the IRR response level, i.e., the number of CA in the relevant dose range, rose with LET, amounting to 2.5-fold for top and bottom values. It logically corresponded to an increase in HRS response dose range at constant dose curve slopes. As for apparent controversy between the IRR CA level and LET_d_ for neutrons and ^12^C at the “tail” (Figure 5), it vanished when we considered track-average LET_t_ of radiations (12 keV/μm for neutrons [25] and 22 keV/μm for carbons). Alternatively, we assessed isoeffective neutron LET_d_ = 25–30 keV/μm. It results from averaging CHO-K1 RBE, as a function of LET for CA [26], over 14.5 MeV neutron dose distribution in LET [27].

**Figure 6:**
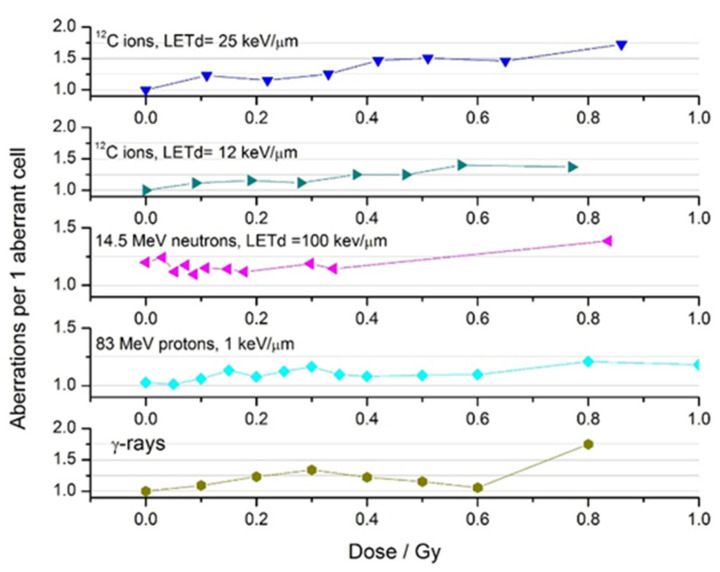
Number of chromosome aberrations per aberrant cell in CHO-K1 cells exposed to low doses of radiations with different LET values.

We analysed the number of CA per 1 aberrant metaphase in the CHO-K1 cell population exposed to different radiations (Figure 6).

One can see that, in the dose range of HRS/IRR response (<0.5–0.6 Gy for ^60^Co γ-rays and protons, <0.4 Gy for neutrons and ^12^C ions), one aberrant cell contained between 1 and 1.25 CA irrespective of LET. (A “hump” in the γ-rays curve was apparently due to chromatid-type aberrations; Table 1).

Data in Table 1 show that HRS/IRR response in the cytogenetic test was provided by a small fraction of the cell population—aberrant cells amounting to ≈10%. The CA increase in the HRS dose range was due to aberrant cell increase rather than CA per cell.

## 3. Discussion

In the present study, we demonstrated a low-dose hypersensitivity and induced radioresistance response using CA induction in stationary-phase Chinese hamster CHO-K1 cells exposed to a scanning proton beam at the Bragg curve plateau and to 14.5 MeV neutrons. The data normalisation per the absorbed radiation dose provided another line of evidence of the above response. Although the radiations had very different dose-averaged LET values, ca. 1 and 100 keV/μm, the phenomena patterns were rather close, viz., a distinct region of HRS response with a steep increase in CA frequency, compared with linear-quadratic extrapolation from higher doses. Another specific region in the dose curve then followed—that of induced radioresistance—where CA yield came to plateau (neutrons) or slowed down (protons) (Figure 1 and Figure 3). It is of importance to note that radiosensitivity, i.e., CA frequency per unit radiation dose, was persistently increased in both dose curves regions until the transition to regular dose dependence, linear–quadratic or linear ones. Therefore, the CA yield also increased when compared with the linear–quadratic or linear prediction (Figure 1 and Figure 3).

Results of the present cytogenetic study, together with those previously published on the observation of HRS/IRR response in plateau-phase CHO-K1 cells exposed to photon and particle radiations with different LET values (Figure 5), enable us to draw further conclusions about LET dependence of the effect. The most prominent one is that the CA frequency level in the IRR region rises in general with radiation LET. We may note that this is in line with a well-known increase in the biological efficiency of radiation with LET. The next two conclusions coincide with those inferred by Marples et al. from HRS/IRR studies on cell survival exposed to low-LET radiation—X-rays, and γ-rays—and medium- and high-LET ones—negative pi-mesons, protons, and neutrons. They stated that “HRS is a ubiquitous response for all radiation qualities” and added, “The transition point (i.e., differential effectiveness of radiation killing per unit dose) between HRS and IRR differs for differing LET radiations” [4] (p. 1311). In our studies, HRS upper border shifted with LET from 0.08 Gy for ^60^Co γ-rays to 0.15 Gy for 14.5 MeV neutrons. The authors also concluded, on the basis of results published up to 2008, that the IRR response is only evident after low and intermediate LET radiation exposures [4] (p. 1311) and that the dose range of IRR response decreases with the increase in LET, up to fully diminishing at high LET [11]. We observed decreases in that dose range, 0.08–0.6 Gy (^60^Co γ-rays), 0.1–0.5 Gy (protons), 0.12–0.35 (carbon ions), and 0.15–0.35 Gy (14.5 MeV neutrons). Data for 14.5 MeV neutrons appear contradictory to some of the above conclusions. Neutron LET_d_ value of ≈100 keV/μm is too high to induce an IRR response according to suggestion in [4]. However, the IRR response do exist and quantitatively is close to that of carbon ions at the Bragg curve “tail” where the LET_d_ value was 25–27 keV/μm.

The reason for the discrepancy between the neutrons’ high LET_d_ value and an intermediate-LET-like biological effect may lie in the spectrum of secondary charged particles produced in tissue by 14.5 MeV neutrons. Partial doses of protons, α-particles, and heavy recoils C, N, and O are 72.9%, 12.4%, and 10.9%, respectively [28]. Corresponding particles LET ranges are approximately 3.5–93 keV/μm, 50–240 keV/μm, and 200–1000 keV/μm. It is the heavy recoils with their LET that give the LET_d_ value of 90–100 keV/μm. However, if we consider the number of particles crossing the cell nucleus rather than deposited energy, we obtain a spectrum-averaged LET value of 12 keV/μm, the so-called track average LET_t_. The numerous protons determine low-neutron LET_t_. We may further suggest that the observed cytogenetic effects at low doses < 0.5 Gy were mainly due to protons, the dose-average LET of which was between 20 and 25 keV/μm [29,30,31]. Cells damaged heavily with high LET α-particles and C, N, and O recoils may escape from analysis due to cell division delay or apoptotic death. Another piece of evidence revealing the proton role in the effect follows from our assessment of 14.7 neutrons effective LET_d_ for CA induction in Chinese hamster cells, which was found to be 25–30 keV/μm. Our putative explanation agrees with the notion that LET, and especially averaged values, is not a good and comprehensive characteristic of radiation quality [32,33,34].

The analysis of the number of CA per one aberrant cell and chromosome-type exchanges fraction in chromosome spectra suggested that lesion repair occurred in the HRS/IRR dose range. It follows from comparing ≈1 CA per aberrant cell with 1 to 10–20 DSB produced in a mammalian cell in the dose range of HRS/IRR response. An increase in chromosome-type exchange fractions resulting from DSB misrepair from ≈20% for carbon ions to 45–50% for γ-rays and protons agrees with the suggestion of simple DSB production in the latter case. Fractions of aberrant cells in the HRS/IRR dose range up to 10% are close to killed cells fractions in survival assays, of 10–20%, i.e., survival levels of 80–90%.

A peculiar shape of the dose dependence of the HRS/IRR phenomenon observed in mammalian cell survival was earlier seen in cytogenetic studies on human lymphocytes. The description of the effect for both tests is actually the same: “The yield [of dicentrics] is very small at 5 rad, then shows a rapid rise between 7.5 and 10 rad, followed by a plateau between 10 and 30 rad and a new rise from 30 rad on” [35] (p. 372). Prior to this publication, Luchnik had proposed a hypothesis about the existence of two types of repair. We continue to cite: ”The “regular” repair occurs during each mitotic cycle and ensures the maintenance of genetic stability. The “emergency” repair is induced by an elevated level of genetic damage. One can speculate that the threshold is produced at doses of radiation where the “regular” repair is already not sufficient but the “emergency” repair is still not induced” [35] (p. 375). Unfortunately, cytogeneticists had paid major attention to the plateau region of the dose curve while overlooking the existence of the HRS region, though they had pointed out “the rapid rise” in dicentrics before the plateau, and that “the number of dicentrics appears to be higher than both theoretical predictions”. However, the similar shape of dose curves for both endpoints is not surprising since the so-called asymmetric types of chromosome aberrations contributed mainly to cell death assessed with clonogenic assay (at least for cells in the G1 or plateau phase). This emphasises the importance of considering the results of the two assays in parallel or concomitantly because the information gained independently might be complementary.

As an example of such a complementary approach, let us consider the possible existence of the low-dose threshold of X-rays for HRS response. All cell survival dose–response data fitted well to the induced-repair model [1] in which HRS response, if existing, started from zero doses, without any threshold. However, Luchnik and Sevan’kaev pointed to it directly in the citation above, placing it between 0.05 and 0.075 Gy. Nearly the same figures follow from the results of multi-lab collaborative studies with human lymphocytes, which have shown a lack of dose–response data in the dose range below 0.02–0.05 Gy [36] and no evidence of HRS up to 0.05 Gy [36,37]. The next dose points in those studies were ≈0.3 Gy, according to Luchnik’s data ([35], Figure 4a). the HRS–IRR response occurred at doses between 0.075 and 0.3 Gy, which were not included in these studies. Therefore, the question of a threshold remained open. It should be noted that Lloyd et al. [37] claimed that linear coefficients in the low dose range studied were consistent with extrapolation from high doses, unlike Luchnik’s finding. This claim agrees with a micro-dosimetric calculation of the mean specific energy z in a cell nucleus of 8 μm diameter which began to linearly increase at doses ≥ 0.0025 Gy after it was constant at lower doses of ^60^Co γ-rays ([9], Figure 1). In a more recent study on dicentric yield in human lymphocytes, the authors did not observe the dependence of chromosome frequency on doses up to 100 mGy [38]. They also did not observe any HRS/IRR response because there were only two dose points. In general, the dose curve for five individuals was linear–quadratic. By contrast, HRS response started already at the dose of 0.05 Gy in cytogenetic studies using micronuclei induction in human fibroblasts, keratinocytes [39], and G2-phase lymphoblastoid cells [40]. However, dose–response curve for total aberrations in G2-phase lymphocytes from donor 1 indicated the apparent threshold somewhere between 0.1 and 0.2 Gy ([40], Figure 1a). Thus, to date, there is no clear evidence for a low border of HRS response in cells exposed to X-rays to be disregarded. It may lie, according to cytogenetic data discussed [35,36,37,41], between 0.05–0.075 Gy and 0.1 Gy (or somewhat higher).

As for other radiations, cytogenetic data give no indication of a low border of HRS response in mammalian cells and dose curves starting from zero doses, or more accurately, from the least doses. In our investigations with CHO-K1 cells, they were 0.05 Gy (protons, this paper); 0.03, 0.05 Gy (neutrons [20]); 0.07 and 0.1 Gy (^12^ C, [24]); and 0.02, 0.03 Gy (protons, fibrosarcoma B14–150 cells [21]). The first dose points were 0.075 Gy (14 MeV neutrons, human melanoma cells) in [14]) and 0.1 Gy (^12^ C ions, HPRT mutation frequency) in [17]. In another multi-lab collaborative study with 14.83 MeV neutrons, in which the studied doses were 3.55, 8.4, 16.4, 24.5, 40.8, 81.1, and 244 mGy, total aberration frequencies increased linearly, starting from 3.55 mGy [31], without any indication of HRS response. In this regard, we should remind the existence of HRS positive/negative patients with cancer, as revealed by Slonina et al. [6], and we could also presume that feature in healthy people. This presumption may partly explain the absence of HRS/IRR response in cells of human origin including lymphocytes, as was observed in several multi-lab studies [31,36,37,42].

The existence of a low-dose threshold—at least for X- or γ-ray exposure—ensues from the Luchnik’s hypothesis about “regular” and “emergency” types of cellular repair (see citation above, this paragraph). To compare quantitatively cytogenetic patterns of HRS/IRR response, it is helpful to apply a piecewise linear fit model of the kind proposed by Geras’kin [43] or separate linear fitting of data in HRS and IRR dose ranges [41].

The results of our investigations with different radiations agree with the assumption made by Marples that HRS/IRR response is seen only after exposure to low/intermediate-LET radiations (with a reservation of about 14 MeV neutrons). Marples had further assumed that there is an upper LET threshold for increased radioresistance detection, using survival assay. The basis for the assumption was the lack of the IRR response in the survival of V-79 cells exposed to d(4)-Be neutrons (LET = 60–70 keV/μm) and Bragg-peak-negative pi mesons (LET = 35–55 keV/μm). However, some articles appeared later in which authors reported HRS/IRR response for 102 keV/μm α-particles [15], and carbon ions with LET values of 45.2 keV/μm [16], 70 keV/μm [17,18], 252 keV/μm [19]. A survival assay was used in all studies, whereas Xue et al. [17] additionally used an HPRT mutation assay. As for the latter study, we may speculate that the effect was due to appreciable low-LET components in high-LET beams with remarkable inhomogeneity for high-LET fractions and homogeneity for low-LET ones, in line with our consideration of 14 MeV neutrons. After X-ray exposure, the IRR response dose range was 0.1–0.5 Gy and 0.12–0.5 Gy for survival and mutation assays, respectively. After exposure to carbon ions (70 keV/μm), range origins shifted to larger doses (0.17 and 0.2 Gy), while range endings did not. Thus, the dose ranges for high-LET ions were shorter than for X-rays, just as in our studies with 14 MeV neutrons.

Identification of DNA DSB as a main cellular radiation lesion that results in radiobiological effects accentuates the role of radiation with various LET values differing in DSB complexity to test conceptions/mechanisms of DNA damage repair. In this regard, mechanistic biophysical modelling of low-dose effects of radiations with different LET values is a perspective tool in the investigation of HRS/IRR phenomena. The Monte Carlo simulation code PARTRAC is a standard of such modelling which considers all stages of radiation effects on subcellular and cellular scales: physical, physiochemical, chemical, biochemical, and biological [44]. In addition to modelling DNA lesions induced by different radiations, it models DSB repair by NHEJ, production of chromosomal aberrations, and cell death. The PARTRAC code is currently being developed to involve radiation-induced effects of protons and light ions at radiotherapy-relevant energies [45]. On the basis of another simulation code Geant4-DNA_2019 similar to PARTRAC, a new version of Geant4-DNA is being developed which additionally takes into account all known DSB repair pathways NHEJ, HR, SSA, alt-NHEJ [46]. However, from the HRS/IRR point of view, the latest proposed mechanisms of low-dose effects should be considered [47]. Another example of less sophisticated modelling is the DNA damage-repair dynamic model for HRS/IRR effects of C. elegans induced by neutron irradiation, developed by Feng et al. [48].

There are suggestions to use the HRS/IRR phenomena in radiation therapy, and reports of such trials in schemes of hyperfractionation with right daily fractions [49]. The goal is either to decrease the total dose to tumours, due to the HRS effect, and consequently to healthy tissues, or to expose healthy tissue at doses in the range of IRR effect, thus sparing it. The latter may be implemented, for example, in multi-directional proton therapy. A certain obstacle in the way of clinical application is the existence of HRS-positive and -negative patients [6], so we need marker(s) of patient HRS/IRR status.

The HRS/IRR phenomenon appears to be many aspects in its manifestations. It was documented in the induction of DNA DSBs, mutations, chromosomal aberrations, micronuclei, and some form of cellular death (reproductive, apoptotic, etc.), using radiation with different LET values. We speculate that its mechanism might involve many aspects as well.

## 4. Materials and Methods

### 4.1. Cell Cultures and Cell Maintenance

Chinese hamster ovary cells CHO-K1 were obtained from the Russian Cell Culture Collection (RCCC) of the Institute of Cytology of the Russian Academy of Sciences. Cell lines were maintained in 25 cm^2^ plastic flasks (Corning) in Ham’s F-12 Nutrient Mixture (CHO-K1) in a humidified 95% air with a 5% CO_2_ incubator at 37 °C. The medium (Paneco, Moscow, Russia) was supplemented with 10% foetal bovine serum (FBS: Biosera, Nuaille, France) and penicillin–streptomycin antibiotics (Paneco, Moscow, Russia). Cells were subcultured from a flask by rinsing and exposing to 0.25% trypsin solution. The number of cells was determined using a cell counter CytoSMART (Corning, Skillman, USA). For all experiments, 3 × 10^5^ CHO-K1 cells were seeded into new 25 cm^2^ plastic flasks, 4–5 days before irradiation, to enable the cultures to be at approximately confluent monolayer and at the stationary growth stage. All flasks were filled with Hanks solution before irradiation.

### 4.2. Irradiations

Irradiations with scanning proton beam (ø 4–7 mm) were performed at the “Prometheus” proton accelerator (“Protom” Ltd., Obninsk, Russia). The proton energy was 83 MeV (LET ~1 keV/μm) under the experimental conditions. Cell monolayers were irradiated at the Bragg curve plateau in the dose range of 0.05–3 Gy. Absorbed doses were measured using a plane-parallel chamber ROOS, 3194. The centre of the sensitive chamber volume was located at the same depth as that of the cell monolayer.

In addition, 14.5 MeV neutron irradiation was carried out at the ING-031 pulsed neutron generator with a sealed tube (VNIIA, The Federal State Unitary Enterprise Dukhov Automatics Research Institute, Moscow, Russia). Pulse duration was 1 μs, pulse frequency was 50 Hz, and neutron yield was 10^9^ s^−1^. In the experiment, cell monolayers were irradiated under conditions of the secondary charged-particle equilibrium in glass Carrel flasks (Ø 4 cm). Briefly, 3 ml of Hanks’ solution was added to each flask to provide these conditions and the required tissue-equivalent liquid thickness (2.5–3 mm). Under those conditions, doses were 0.1–0.9 Gy, and LET_d_ was estimated to be ~100 keV/μm. Absorbed doses were measured using a Unidos dosimetry system (PTW, Freiburg, Germany).

A U-70 accelerator (IHEP, Protvino, Russia) generated carbon ions, with an initial energy of 454 MeV/amu. Cells were irradiated at the proximal (a depth of ~10 cm in a water phantom, LET_d_~10–12 keV/μm) and the distal region of the pristine Bragg curve (~1 cm behind the Bragg peak, LET_d_~25–27 keV/μm) in the dose range of 0.05–2.5 Gy. Physical and dosimetry studies were published elsewhere [50,51,52].

The cytogenetic efficiency of protons, neutrons, and carbon ions was compared with the action of standard γ-radiation. The flasks with cell monolayers were irradiated at a gamma unit with a ^60^Co source (E = 1.25 MeV) under electronic equilibrium conditions. The dose range was 0.08–3.19 Gy, and the dose rate was 0.05 Gy/min. The dose rates for all particle radiations were 0.03–0.06 Gy/min.

All of the irradiations were performed at room temperature. Two or more independent experiments were made with each irradiation (and LET). The dosimetry error did not exceed 10% (*p* = 0.95).

### 4.3. Metaphase Analysis

After irradiation, flasks with cells were transported to the laboratory on melting ice to slow down the recovery processes. Then, cells were removed from the growth surface with a trypsin solution, reseeded in a fresh medium supplemented with 20% of FBS, and incubated during 24–25 h in a CO_2_ incubator at 37 °C and 5% CO_2_; two hours before fixation, colchicine was added. Slides of the first mitosis metaphases were prepared using standard procedures [53]. Structural chromosome aberrations of all types visible by Giemsa staining were scored, both chromosome and chromatid types. We assigned one paired fragment to each dicentric or centric ring and scored excess fragments as terminal deletions. In the article we name terminal deletions as paired fragments in Table A1, Table A2, Table A3, Table A4 and Table A5 of Appendix A. For each experimental point, 600 to 2400 metaphases were analysed.

### 4.4. Statistical Analysis

Standard methods of statistics implemented in Microsoft Excel and OriginLab software were used. The experimental data were fitted to the linear–quadratic model Equation (1), and to its modification by Marples and Joiner in the form of the induced-repair model (IR model, (2)) [1] that we described in [21] as follows:


(1) $$Y=\alpha D+\beta D^{\;2}$$

(2) $$Y=\alpha [1+\left(\alpha_{s}/\alpha -1)exp\left(–D/D_{c}\;\right)\right]D+\beta D^{\;2}$$

where *Y*—the quantity of total of chromosome aberrations per cell;
*D*—the absorbed dose, Gy;
α, Gy^−1^, and *β*, Gy^−2^—linear and quadratic regression coefficients, respectively;
*α_s_*—the initial slope of the dose curve derived from the response at very low doses;
*D_c_*—the “transition” dose at which the radioresistance induction is 63% of the maximum value.

## Figures and Tables

**Table 1:** Cytogenetic parameters of CHO-K1 cell population exposed to low doses of radiations with different LET values.

Dose, Gy	Cells Scored	Aberrant Cells	Aberrant. Cells, %	Total CA	CA/Aberrant Cell	Chromosome Type/Total CA	Chromosome Exchanges/Chromosome Type Total
^60^Co γ-rays							
0	1500	1	0.1	1	1.00	1	1.00
0.1	1700	65	3.8	71	1.09	0.92	0.36
0.2	2000	69	3.5	85	1.23	0.87	0.45
0.3	1800	56	3.1	75	1.34	0.87	0.35
0.4	1600	63	3.9	77	1.22	0.88	0.32
0.5	1900	91	4.8	105	1.15	0.89	0.25
0.6	1200	71	5.9	75	1.06	0.99	0.33
0.8	1400	140	10.0	245	1.75	0.7	0.41
83 MeV protons							
0	1400	37	2.6	38	1.03	0.84	0.69
0.05	1600	80	5.0	81	1.01	0.91	0.50
0.1	2200	169	7.7	179	1.06	0.90	0.42
0.15	2400	190	7.9	215	1.13	0.91	0.45
0.2	2600	205	7.9	221	1.08	0.89	0.43
0.25	2400	208	8.7	234	1.13	0.90	0.45
0.3	2400	213	8.9	248	1.16	0.77	0.43
0.35	1800	155	8.6	170	1.10	0.86	0.46
0.4	2400	211	8.8	228	1.08	0.91	0.37
0.5	2400	221	9.2	241	1.09	0.88	0.39
0.6	2000	262	13.1	287	1.10	0.93	0.55
0.8	1600	263	16.4	318	1.21	0.87	0.57
1	600	99	16.5	117	1.18	0.91	0.50
14.5 MeV neutrons							
0.00	1500	5	0.33	6	1.20	0	0
0.03	1500	33	2.2	41	1.24	0.63	0.29
0.05	1000	34	3.4	38	1.12	0.84	0.34
0.07	1600	40	2.5	47	1.18	0.73	0.36
0.09	1200	52	4.3	57	1.10	0.90	0.19
0.11	1500	80	5.3	92	1.15	0.92	0.18
0.15	1200	113	9.4	129	1.14	0.93	0.27
0.18	800	60	7.5	67	1.12	0.90	0.33
0.30	800	64	8.0	76	1.19	0.83	0.43
0.34	800	83	10.4	95	1.14	0.79	0.33
0.84	1000	166	16.6	230	1.39	0.91	0.33
^12^C, plateau							
0	1600	6	0.38	6	1.00	0.33	0
0.09	2500	97	3.9	108	1.11	0.79	0.33
0.19	1800	84	4.7	97	1.15	0.79	0.27
0.28	1500	93	6.2	104	1.12	0.88	0.17
0.38	1000	92	9.2	115	1.25	0.89	0.23
0.47	800	113	14.1	141	1.25	0.82	0.17
0.57	600	125	20.8	175	1.40	0.9	0.12
0.77	800	214	35.7	416	1.37	0.84	0.23
^12^C, “tail”							
0	1600	6	0.38	6	1.00	0.33	1.00
0.12	1100	100	9.1	123	1.23	0.79	0.18
0.23	1400	111	7.9	128	1.15	0.76	0.23
0.35	1400	154	11.0	193	1.25	0.77	0.21
0.45	900	160	17.8	235	1.47	0.82	0.19
0.57	800	198	24.7	298	1.51	0.82	0.21
0.70	600	203	33.8	296	1.46	0.77	0.16
0.93	600	241	40.2	416	1.73	0.74	0.26

Beyond the upper border of IRR response number of CA per 1 aberrant cell begins to rise with the dose increase. Experimental results show that 1 Gy of X-rays produces from 20 to 40 initial γH2AX foci per mammalian cell as was reviewed in [5]. Assuming that this focus numbers correspond to DSBs we can estimate that in the dose range of HRS/IRR response (0.05 ÷ 0.5 Gy) low-LET radiations (γ-rays, high-energy protons) produce ≈1 to 10–20 DSBs per cell. Comparing nearly constant 1 CA per cell with growing DSB numbers thus indicates increasing with dose DSB repair. Another evidence of DSB repair is the production of chromosome-type exchanges, which result from DSB misrepair, e.g., by NHEJ (nonhomologous end-joining, shown in the last column in Table 1). It is interesting that the exchange fraction increases with LET decrease, from ≈20% for ^12^ C ions to 45–50% for γ-rays and protons. This suggests a simpler DSB in the latter cases. Furthermore, 14.5 MeV neutrons exhibit an intermediate behaviour.

## Abbreviations

CA	Chromosomal aberrations
LET	Linear energy transfer
HRS	Hyper-radiosensitive
IRR	Induced radioresistance
DSB	Double-strand breaks
LQ	Linear quadratic
IR	Induced repair
FBS	Fetal bovine serum
NHEJ	Nonhomologous end joining
HR	Homologous recombination
SSA	Single-strand annealing
Alt-NHEJ	Alternative nonhomologous end joining

## Appendix A

This appendix contains details of the chromosomal analysis of CHO-K1 exposed to ^60^Co γ-rays, 83 MeV protons, 14.5 MeV neutrons, and ^12^C ions beam at proximal (plateau) and distal (“tail”) regions of the Bragg curve.

**Table A1:** Frequencies of chromosome aberrations induced in CHO-K1 cells by low doses of ^60^Co γ-rays.

D, Gy	Number of Cells Scored	All Aberrations/100 Cells	Number of Aberrations per 100 Cells							
			Chromosome Type				Chromatid Type			
			Paired Fragments	Dicentrics and Centric Rings	Interstitial Deletions	Total	Deletions	Isochromatid Deletions	Isochromatid Exchanges	Total
0	1400	0.14 ± 0.10	–	0.07	–	0.07	–	–	0.07	0.07
0.08	200	7.65 ± 1.40	4.59 ± 1.09	2.55		7.14	0.51	–	–	0.51
0.10	1700	4.41 ± 0.52	2.24 ± 0.38	1.47	0.35	4.06	0.06	0.06	0.23	0.35
0.15	200	4.00 ± 2.00	4.00 ± 2.00	–	–	4.00	–	–	–	–
0.20	2000	4.25 ± 0.50	2.40 ± 0.38	1.10	0.20	3.70	–	0.05	0.50	0.55
0.30	2050	4.55 ± 0.51	2.68 ± 0.39	1.07	0.20	3.95	0.05	0.10	0.45	0.60
0.40	1600	4.81 ± 0.58	3.19 ± 0.47	0.87	0.19	4.25	0.06	0.19	0.31	0.56
0.50	1900	5.52 ± 0.53	3.26 ± 0.41	1.21	0.42	4.89	0.26	0.16	0.21	0.63
0.60	1200	6.25 ± 0.74	3.67 ± 0.55	1.50	1.00	6.17	–	–	0.08	0.08
0.80	1400	17.49 ± 1.28	9.14 ± 0.96	2.64	0.43	12.21	0.71	3.14	1.43	5.28

**Table A2:** Frequencies of chromosome aberrations induced in CHO-K1 cells by low doses of 83 MeV protons.

D, Gy	Number of Cells Scored	All Aberrations/100 Cells	Number of Aberrations per 100 Cells							
			Chromosome Type				Chromatid Type			
			Paired Fragments	Dicentrics and Centric Rings	Interstitial Deletions	Total	Deletions	Isochromatid Deletions	Isochromatid Exchanges	Total
0.00	1400	2.71 ± 0.44	0.71 ± 0.22	1.57	0.00	2.29	0.00	0.00	0.43	0.43
0.05	1600	5.06 ± 0.55	2.31 ± 0.44	2.19	0.13	4.63	0.06	0.00	0.38	0.44
0.1	2200	8.14 ± 0.49	4.27 ± 0.56	2.91	0.14	7.32	0.41	0.00	0.41	0.82
0.15	2400	8.96 ± 0.67	4.50 ± 0.53	3.58	0.08	8.17	0.38	0.00	0.42	0.79
0.2	2600	8.50 ± 0.59	4.35 ± 0.43	3.15	0.08	7.58	0.19	0.00	0.73	0.92
0.25	2400	9.75 ± 0.68	4.88 ± 0.64	3.84	0.08	8.79	0.29	0.00	0.67	0.96
0.3	2400	10.33 ± 0.73	4.58 ± 0.72	3.17	0.25	8.00	0.67	0.04	1.62	2.33
0.35	1800	9.44 ± 0.76	4.44 ± 0.71	3.67	0.06	8.17	0.28	0.00	1.00	1.28
0.4	2400	9.50 ± 0.65	5.42 ± 0.71	3.12	0.08	8.63	0.33	0.00	0.54	0.88
0.5	2400	10.04 ± 0.67	5.42 ± 0.64	3.33	0.08	8.83	0.46	0.04	0.71	1.21
0.6	2000	14.35 ± 0.87	5.95 ± 0.75	7.25	0.15	13.35	0.35	0.00	0.65	1.00
0.8	1600	19.88 ± 1.22	7.50 ± 0.80	8.69	1.06	17.25	0.63	0.19	1.82	2.63
1	600	19.50 ± 1.92	8.83 ± 1.32	8.00	1.00	17.83	0.50	0.00	1.17	1.67

**Table A3:** Frequencies of chromosome aberrations induced in CHO-K1 cells by low doses of 14.5 MeV neutrons.

D, Gy	Number of Cells Scored	All Aberrations/100 Cells	Number of Aberrations per 100 Cells							
			Chromosome Type				Chromatid Type			
			Paired Fragments	Dicentrics and Centric Rings	Interstitial Deletions	Total	Deletions	Isochromatid Deletions	Isochromatid Exchanges	Total
0.00	1500	0.4 ± 0.28	–	–	–	–	0.1	0.1	0.2	0.4
0.03	1500	2.7 ± 0.51	1.2 ± 0.29	0.4	0.1	1.7	0.1	0.1	0.8	1
0.05	1000	3.8 ± 0.67	2.1 ± 0.52	1.1	–	3.2	0.2	0.1	0.3	0.6
0.07	800	4.5 ± 0.91	2.1 ± 0.65	1.1	0.1	3.3	0.3	–	0.9	1.2
0.09	1200	4.8 ± 0.67	3.5 ± 0.58	0.6	0.2	4.3	0.3	0.1	0.1	0.5
0.11	1500	6.2 ± 0.74	4.7 ± 0.63	0.9	0.1	5.7	0.3	–	0.2	0.5
0.15	1200	10.8 ± 1.02	7.3 ± 0.83	2.4	0.3	10	0.1	0.2	0.5	0.8
0.18	800	8.4 ± 1.1	5.1 ± 0.84	1.6	0.9	7.6	0.1	0.4	0.3	0.8
0.30	800	9.5 ± 1.21	4.5 ± 0.81	3.4	–	7.9	0.2	–	1.4	1.6
0.34	800	11.9 ± 1.33	6.3 ± 0.89	2.6	0.5	9.4	1.5	–	1	2.5
0.84	800	23.0 ± 0.92	14.1 ± 0.38	5.5	1.4	21	0.4	0.2	1.4	2

**Table A4:** Frequencies of chromosome aberrations induced in CHO-K1 cells by low doses of ^12^ C ions at Bragg curve plateau.

D, Gy	Number of Cells Scored	All Aberrations/100 Cells	Number of Aberrations per 100 Cells							
			Chromosome Type				Chromatid Type			
			Paired Fragments	Dicentrics and Centric Rings	Interstitial Deletions	Total	Deletions	Isochromatid Deletions	Isochromatid Exchanges	Total
0	1600	0.38 ± 0.03	0.13 ± 0.09	–	–	0.13	0.06	–	0.19	0.25
0.07	1400	5.14 ± 0.63	3.43 ± 0.53	0.42	–	3.85	0.29	0.07	0.93	1.29
0.09	2500	4.32 ± 0.46	2.16 ± 0.31	0.96	0.12	3.24	0.04	0.04	1	1.08
0.19	1800	5.39 ± 0.61	5.30 ± 0.44	1.40	0.10	6.80	0.60	–	0.70	1.30
0.28	1500	6.93 ± 0.74	2.94 ± 0.60	0.94	0.17	4.05	0.33	0.06	0.94	1.33
0.38	1000	11.50 ± 1.26	5.06 ± 0.98	0.87	0.13	6.06	0.20	–	0.67	0.87
0.47	800	17.63 ± 1.74	7.90 ± 1.34	2.20	0.10	10.20	0.40	–	0.90	1.30
0.57	600	29.16 ± 2.75	11.75 ± 2.41	2.00	0.38	14.13	1.00	–	2.50	3.50
0.60	900	28.00 ± 2.18	19.44 ± 1.86	3.33	0.56	23.33	0.78	–	3.89	4.67
0.77	800	36.75 ± 2.54	23.63 ± 1.96	6.50	0.50	30.63	1.50	0.50	4.13	6.13

**Table A5:** Frequencies of chromosome aberrations induced in CHO-K1 cells by low doses of ^12^ C ions at Bragg curve “tail”.

D, Gy	Number of Cells Scored	All Aberrations/100 Cells	Number of Aberrations per 100 Cells							
			Chromosome Type				Chromatid Type			
			Paired Fragments	Dicentrics and Centric Rings	Interstitial Deletions	Total	Deletions	Isochromatid Deletions	Isochromatid Exchanges	Total
0	1600	0.38 ± 0.03	0.13 ± 0.09	–	–	0.13	0.06	–	0.19	0.25
0.12	1100	11.18 ± 1.35	7.09 ± 0.88	1.46	0.09	8.64	0.45	0.18	1.91	2.54
0.23	1400	9.14 ± 0.89	5.29 ± 0.64	1.57	–	6.86	0.57	0.07	1.64	2.28
0.35	1400	13.78 ± 1.15	8.64 ± 0.91	2.07	0.22	10.93	1.00	0.21	1.64	2.85
0.45	900	26.11 ± 2.07	17.45 ± 1.73	4.0	–	21.45	1.22	–	3.44	4.66
0.57	800	37.25 ± 2.70	23.50 ± 2.23	6.25	0.13	29.88	1.25	0.37	5.75	7.37
0.70	600	49.33 ± 3.38	31.67 ± 2.69	5.16	0.83	37.66	3.50	0.50	7.67	11.67
0.93	600	69.33 ± 4.34	42.33 ± 3.30	8.17	0.33	50.83	3.0	0.17	15.33	18.50
